# Challenging Paradigms Through Ecological Neuroscience: Lessons From Visual Models

**DOI:** 10.3389/fnins.2021.758388

**Published:** 2021-11-10

**Authors:** Giuliana Bucci-Mansilla, Sergio Vicencio-Jimenez, Miguel Concha-Miranda, Rocio Loyola-Navarro

**Affiliations:** ^1^Neurosystems Laboratory, Department of Neuroscience and Biomedical Neuroscience Institute, Faculty of Medicine, Universidad de Chile, Santiago, Chile; ^2^Ecological Cognitive Neuroscience Group, Santiago, Chile; ^3^The Center for Hearing and Balance, Otolaryngology-Head and Neck Surgery, Johns Hopkins University School of Medicine, Baltimore, MD, United States; ^4^Bernstein Center for Computational Neuroscience Berlin, Humboldt-Universität zu Berlin, Berlin, Germany; ^5^Neuroscience, Cognition and Educational Lab, Center for Advanced Research in Education, Institute of Education, Universidad de Chile, Santiago, Chile; ^6^Departamento de Educación Diferencial, Facultad de Filosofía y Educación, Universidad Metropolitana de Ciencias de la Educación, Santiago, Chile

**Keywords:** ecological neuroscience, perception, cognition, sensorimotor loop, active sensing, dynamic perception, neural coding

## Introduction

Perhaps the ultimate goal of neuroscience is to generate models and theories to explain how our nervous system works in its natural environment. To a large extent, this goal has been pursued with experimental approaches that have a high degree of control of experimental variables, isolating the one of interest while trying to keep all others constant. In the field of perceptual neuroscience, this approach has made it possible to study the brain's responses to specific stimuli in a precise manner, leading to the development of explanatory models. However, a high degree of variable control is not without its drawbacks. It has been proposed that when experimental control is taken to an extreme level, it is possible to generate conditions so specific that experiments cannot be replicated by other researchers (Voelkl et al., 2020), generating a kind of experimental endemism. And even when they are replicable, it is difficult to draw conclusions that can be extrapolated to behavior and brain function in nature. In fact, we believe that the greatest risk lies in that evidence obtained under high variable control may lead to models that do not reflect natural brain functioning.

This is how the significance of ecological validity becomes evident. Ecological validity aims to generalize the findings outside the laboratory by designing experiments that resemble the natural conditions where the organism develops and behaves (Shamay-Tsoory and Mendelsohn, 2019). But this statement leaves considerable room for interpretation (Holleman et al., 2020). Therefore, it is important to emphasize that for an experiment to be ecologically valid, it is not necessary to recreate “real life” in the laboratory, but rather capture the necessary features for the conclusions of the experiment to be extrapolated to the phenomenon studied. These features depend on each question and should represent the essential elements that the phenomenon has in the real world.

Therefore, it is relevant to ask how many of our current models have been generated with experiments that are not capturing these necessary features. We suspect that ideas deeply embedded in everyday discourse in neuroscience, such as the neural code, have been established with evidence obtained mostly under highly controlled conditions and low ecological validity. For that reason, it is necessary to challenge them in new, more ecological experimental contexts.

## Moving Toward Ecological Models

In recent years, technological advances have improved our ability to measure and analyze multiple complex variables simultaneously, opening the possibility of studying brain function in ecologically valid contexts. This provides an opportunity to test the paradigms and explanatory metaphors that were established in controlled environments.

The opportunity presented requires considering the fundamental characteristics of the phenomenon we want to study. Since these characteristics depend on each question, first we must identify the phenomenon and determine the essential elements of it. For the case of the neuroscience of perception, these characteristics should include that: it is multisensory, occurs in an awake animal, capable of interacting and moving with purpose. Also, they should consider the particularities of each organism. A primate is “more” visual than a mouse, which navigates relatively more with the tactile sense of its vibrissae than with its eyes alone.

In practice, applying all these features would be a huge challenge, as it would be extremely difficult analyzing these results. Therefore, there is a tradeoff between the degree of ecological validity and the control over our variables. The degree of ecological validity will depend on how many of these features can be implemented in each experiment.

An excellent example of balance between ecological validity and variable control is the research carried out by Wal et al. (2021). Authors investigated how behavioral demands could influence the selectivity to visual stimuli of cortical neurons. They studied awake head-fixed mice in a task where they had to choose between two visual signals for a reward. They also recorded single-neuron activity in the primary visual (V1) or anterior cingulate cortices (ACC) and monitored eye position by eye-tracking. The animal's voluntary movement initiated each trial, resembling their natural exploratory behavior. They found selectivity in V1 neurons during cue evaluation, which arose in response to behavioral demands because the same neurons could not discriminate the cues in sensory control measurements. This design had considerable ecological validity while retaining variable control. This can be seen in that the visual stimuli were simple (non-natural) but context dependent and considered the motor (eye position) influence on them. And also, in that the animals were head-fixed, but their voluntary locomotor activity determined the onset of trials.

Nevertheless, there are more ecologically valid approaches where animals are allowed to freely move while having their eye movements recorded (Wallace et al., 2013; Meyer et al., 2018; Sattler and Wehr, 2020). However, in these experiments, the reconstruction of eye movements and electrophysiological recording is more challenging. In freely moving experiments the stimuli reaching the retina are variable, making any cortical receptive field (RF) estimation difficult. In fact, to our knowledge, this has yet to be implemented. On the other hand, it is possible to reconstruct RF in animals with restricted movement (or anesthetized), but the ecological validity is undermined since the brain is studied as having only task-evoked responsive activity (Raichle, 2010). Most neuroscience research falls into this last category, which has conditioned our results and biased the metaphors we use to communicate how the brain works. For example, the idea of viewing the nervous system signals as a symbolic code originated largely from Adrian's work on sensory neurons (Adrian and Zotterman, 1926; Garson, 2019) performed in a preparation of frog muscle attached to its innervating nerve fiber.

## Challenging Paradigms

The stimuli used to study perception are also relevant, since the degree of similarity with the natural stimulus may affect the ecological validity of the experiment (Shamay-Tsoory and Mendelsohn, 2019; Fan et al., 2021). Classic controlled-reductionist stimuli are designed to isolate a handful of intuitive variables, such as luminance or color. This allows separation of brain activity related to relevant stimuli (i.e., the signal) from noise (i.e., the activity of other processes), but conflicts with real-life stimuli where all these features are intertwined, making stimulus and noise part of the same signal (Nastase et al., 2020). This makes extremely complex generating stimuli that capture the environment's characteristics. For example, macaques present different gaze dynamics when looking at a real conspecific vs. a static image of it (Dal Monte et al., 2016). This highlights that even stimuli that seem like natural ones, can produce different behaviors (and probably different brain activity), stressing the point that neuroscientific models should be tested under ecologically valid experiments.

Predicting neuronal responses from natural scenes in highly controlled experiments is possible at the retina (Schottdorf and Lee, 2021), but at other levels (such as V1) is challenging as complex contextual information affects cortical responses (Koay et al., 2020). This highlights the importance of noting the animal's internal state in how the brain processes such visual stimuli. In this sense, Wal et al. (2021) presented two visual cues that provided equivalent stimulation to V1 neuron's RF. Their experiment showed that neuronal activity in response to the two cues was equivalent without a rewarding context. Interestingly, when one of the cues was associated with a reward, V1 neural activity was sufficiently different to discriminate between both visual cues. Hence, the context under which the animal was interacting with its environment proved crucial in differentiating the stimuli. Such findings make it difficult to defend the idea that just the external stimuli observed by the mouse is causing the neuronal activity of V1, or that these are being represented in it. In this sense, two key components of the neuronal coding concept (representation and causality) (Brette, 2018) are not being met. This is especially interesting considering that such conditions do hold for experiments in anesthetized animals using stimuli similar to those used by Wal et al.

By considering additional variables as part of the signal reaching V1, it has been shown that its activity not only depends on the sensory signal coming from the retina (Chubykin et al., 2013; Stringer et al., 2019; Parker et al., 2020; Schneider, 2020). V1's response is shaped by activity coming within the brain, originating from the subject itself (Zipser et al., 1996; Womelsdorf et al., 2008; Gilbert and Li, 2013). This area is modulated by regions linked to reward-seeking like the ACC (Zhang et al., 2014; Fiser et al., 2016), as well as areas associated with eye movement (Itokazu et al., 2018) and locomotion (Keller et al., 2012; Saleem et al., 2013; Leinweber et al., 2017). This highlights that there is no clear correspondence between the visual stimulus and the activity of the V1 neurons that supposedly encode it. Instead, their activity is better aligned with the animal's context and behavior (Chubykin et al., 2013; Leinweber et al., 2017; Huda et al., 2020; Wal et al., 2021). In other words, if we did not know the context associated with the stimulus (such as the mouse eye position, or its goal-oriented behavior), it would not be possible to determine the nature of the stimulus by just looking at the brain activity. Hence, it would not be possible to decode it.

When brain functioning is considered a circular and dynamic relationship between the motor system, environment and sensory systems, even the activity of primary areas does not statically correspond to external stimuli. For instance, although classic approaches saw eye movements as a variable that needed to be cleared from the signal, it has been well-established that sensation involves the movement of the sensory surface (O'Regan and Noë, 2001; Ahissar and Assa, 2016), thus occurring in a closed-loop sensorimotor scheme. In natural visual perception, objects are scanned by successive fixations joined by eye movements (Berger et al., 2012). Overlooking that animals actively control their visual stimulation by shifting the retina's position constrains our understanding of the brain's mechanism to construe visual perception. For example, in Wal et al., neural responses were more variable when eye position was not controlled, reducing the number of neurons that responded differentially to the two stimuli.

Complementarily, when an animal actively moves through its environment, it also exposes its retina to sensory changes. Leinweber et al. (2017) showed that V1 activity is further modulated by locomotion, interpreted as a prediction signal of visual flow based on motor output from the secondary motor cortex. Thus, it is also relevant to consider locomotion as part of the signal instead of a variable that needs to be cleared. Wal et al. also considered the animal's locomotion in their analysis by including two critical design features: (i) task self-initiation and (ii) analyzing the data in time windows where the animal's motion was equivalent. As with eye movements, this allowed V1 recordings to be made under conditions that considered the sensorimotor state of the animal with its environment. Thus, external visual stimuli that presumably generated the same stimulation in the neural RFs, evoked differential neuronal responses because the overall state of the animal was different when facing those stimuli.

## Final Considerations

All the above shows that the work of Wal et al. is an excellent example of an experimental design that can lead us toward more ecological neuroscience models, where brain function is studied dynamically and in correspondence with the organism's relationship with its environment. This study illustrates that the activity of sensory neurons does not correspond unambiguously with visual signals. It also indicates that, without knowing the animal's context, one could not identify what it was seeing. It further suggests that the systems associated with perception are built from relationships between sensory signals and the organism's actions. This implies that, for developing explanatory models of perception under natural conditions, evidence obtained by experiments with extreme variable control (as in anesthetized animals) is of little use and, perhaps, has even been counterproductive. Given all the above, evidence from ecological neuroscience paradigms supports the idea that traditional neural coding is becoming an insufficient explanatory metaphor. We might need to replace it with closed-loop sensorimotor control models (Brette, 2018) to open new ways of understanding the brain.

## Author Contributions

RL-N, GB-M, SV-J, and MC-M: original idea, first draft, and manuscript comments and edition. RL-N, GB-M, and SV-J: final draft. RL-N and GB-M: funding acquisition. All authors contributed to the article and approved the submitted version.

## Funding

This work was funded by the Millennium Scientific Initiative (Iniciativa Científica Milenio, ICM; Grant Number ICM09_015). Support from ANID/PIA/Basal Funds for Centers of Excellence FB0003 is gratefully acknowledged.

## Conflict of Interest

The authors declare that the research was conducted in the absence of any commercial or financial relationships that could be construed as a potential conflict of interest.

## Publisher's Note

All claims expressed in this article are solely those of the authors and do not necessarily represent those of their affiliated organizations, or those of the publisher, the editors and the reviewers. Any product that may be evaluated in this article, or claim that may be made by its manufacturer, is not guaranteed or endorsed by the publisher.
